# Practice-changing radiation therapy trials for the treatment of cancer: where are we 150 years after the birth of Marie Curie?

**DOI:** 10.1038/s41416-018-0201-z

**Published:** 2018-07-31

**Authors:** Mareike K. Thompson, Philip Poortmans, Anthony J. Chalmers, Corinne Faivre-Finn, Emma Hall, Robert A. Huddart, Yolande Lievens, David Sebag-Montefiore, Charlotte E. Coles

**Affiliations:** 1 Department of Oncology, Cambridge University Hospitals NHS Foundation Trust, Cambridge, CB2 0QQ UK; 2 Institut Curie, 75248 Paris, France; 3 Institute of Cancer Sciences, University of Glasgow, Glasgow, G61 1QH UK; 4 Division of Cancer Sciences, University of Manchester; The Christie NHS Foundation Trust, Manchester, M20 4BX UK; 5 Clinical Trials and Statistics Unit, The Institute of Cancer Research, Sutton London, SM2 5NG UK; 6 Section of Radiotherapy and Imaging, The Institute of Cancer Research, London, SM2 5NG UK; 7 Department of Radiation Oncology, Ghent University Hospital and Ghent University, C. Heymanslaan, 9000 Ghent, Belgium; 8 Radiotherapy Research Group, Leeds Institute of Cancer and Pathology, University of Leeds; Leeds Cancer Centre, St James’s University Hospitals, Leeds, LS9 7TF UK; 9 Department of Oncology, University of Cambridge, Cambridge, CB2 0QQ UK

**Keywords:** Radiotherapy, Breast cancer, Lung cancer, Prostate cancer

## Abstract

As we mark 150 years since the birth of Marie Curie, we reflect on the global advances made in radiation oncology and the current status of radiation therapy (RT) research. Large-scale international RT clinical trials have been fundamental in driving evidence-based change and have served to improve cancer management and to reduce side effects. Radiation therapy trials have also improved practice by increasing quality assurance and consistency in treatment protocols across multiple centres. This review summarises some of the key RT practice-changing clinical trials over the last two decades, in four common cancer sites for which RT is a crucial component of curative treatment: breast, lung, urological and lower gastro-intestinal cancer. We highlight the global inequality in access to RT, and the work of international organisations, such as the International Atomic Energy Agency (IAEA), the European SocieTy for Radiotherapy and Oncology (ESTRO), and the United Kingdom National Cancer Research Institute Clinical and Translational Radiotherapy Research Working Group (CTRad), that aim to improve access to RT and facilitate radiation research. We discuss some emerging RT technologies including proton beam therapy and magnetic resonance linear accelerators and predict likely future directions in clinical RT research.

## Introduction

Born Maria Salomea Skłodowska on 7 November 1867 in Warsaw, Poland, Marie Curie later went on to discover both polonium and radium, and won two Nobel prizes for her work on radioactive substances. Later, during the First World War, she set up a fleet of mobile X-ray units, popularly called ‘the little Curies’, to enable military doctors to locate and remove shrapnel from soldiers’ wounds at the front line. After the war, she was instrumental in founding the Institut du Radium in Paris, which later became part of the Institut Curie, now a world-leading oncology research centre. To mark the 150th anniversary of her birth, we reflect on the global advances made in radiation oncology, focussing on radiation therapy (RT) practice-changing trials over the last two decades. In addition, we discuss global inequalities in access to RT and highlight possible future directions of clinical RT research.

## RT is a crucial and cost-effective cancer treatment

Systemic therapy may mistakenly be considered the mainstay of curative oncological treatment, perhaps due to its high profile in the media. In fact, 40% of patients who are cured of cancer will receive RT as part of their management, and around 50% of cancer patients will require RT at some point during their treatment in high as well as low and middle-income countries.^1–4^ Despite representing a large proportion of cancer treatment, RT accounts for only 5% of the national cancer budget in both the UK and Sweden.^3,5^ By contrast, the European Union average proportion of total oncology expenditure spent on cancer drugs has increased from 12% in 2005 to 23% in 2014.^6^ In addition to its curative potential, RT also has a key role in the palliation of symptoms including pain, bleeding and nerve compression, as well as in curative intent treatments.

RT comprises multiple different treatment modalities, including external beam therapy (encompassing photons, electrons, protons and other particles) and internal/surface treatment (brachytherapy and radiopharmaceuticals). The most widely used modality is megavoltage photon therapy, which is a form of high-energy electromagnetic radiation produced by a linear accelerator. Megavoltage photons have a range of tissue penetration, which allows treatment of deeper internal body structures, such as pelvic organs and lung tumours. Other forms of external beam therapy are orthovoltage photons, which have shallower tissue penetration and are useful for treating skin and soft tissue; and electrons, which also have a short range of tissue penetration but a different dose distribution to orthovoltage photons, and are used mainly for treating skin and superficial tumours. Proton beam therapy is an emerging form of external beam therapy, which has a peak of dose deposition at a sharply defined point (the ‘Bragg peak’) and as such has potential for a much lower dose to nearby critical organs. Internal RT uses very short-range radiation from radioactive sources delivered inside the body. This can be solid sources placed during a surgical procedure or on the body surface, as in brachytherapy for prostate or cervical cancer. Alternatively, unsealed sources may be used, for example radiopharmaceutical injections, which are preferentially taken up by cancer tissues.

**Fig. 1:**
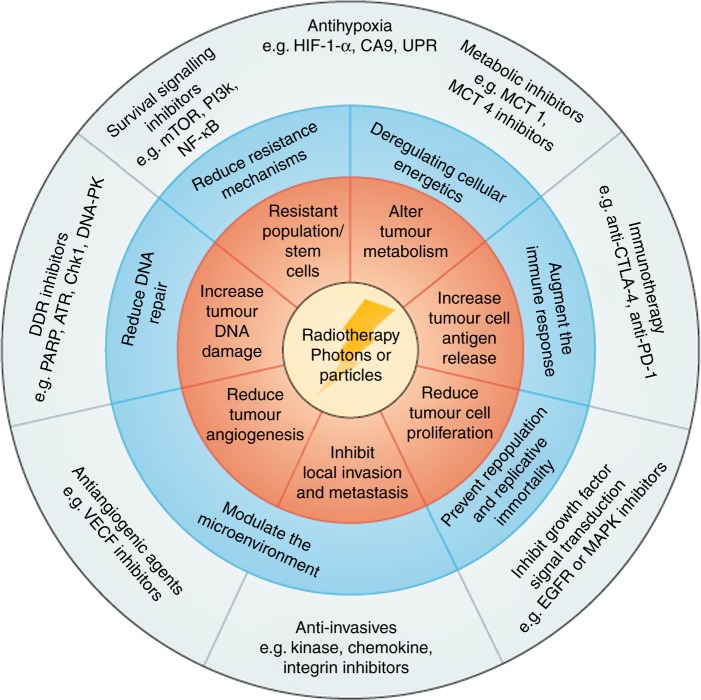
RT has great potential to be combined with multiple classes of novel drugs. Reprinted from Nature Reviews Clinical Oncology.^11^

RT has dramatically improved both technologically and in terms of clinical outcomes; less than two decades ago, RT was mostly given as simple ‘fields’, which are square/rectangular beams, with minimal imaging guidance. Now, it is increasingly common to deliver highly targeted image-guided treatment with intensity modulated RT (IMRT). This enables dose reduction to the surrounding normal structures, thereby minimising toxicity, and facilitates dose escalation to the tumour, thereby maximising cancer control.^7,8^ Another new technique is stereotactic body RT (SBRT), which allows the administration of very high doses of precision radiation in a small number of treatments (fractions).^9^ This is currently used for some primary lung and brain tumours, but also raises the tantalising question of whether patients with a limited number of metastases (‘oligometastatic disease’) could also be cured using this new technology, in combination with systemic therapy. Finally, the mechanistic understanding of how RT interacts with cancerous and normal tissue at a cellular and molecular level is moving at a rapid pace; as a result, exciting opportunities are now arising for investigating RT in combination with novel drugs, such as immunotherapy and DNA damage response inhibitors^10,11^ (Fig. 1).

RT practice has historically varied greatly across international and national institutions, and even within individual centres. Over recent years, a growing focus on evidence-based medicine has led to an increasing number of RT clinical trials that aim to improve patient outcomes by improving overall survival and local tumour control, and/or reducing (often permanent) side effects. Secondary but important benefits of these practice-changing clinical trials include improvements in RT quality and consistency across multiple centres. This manuscript aims to highlight to a non-specialist audience the results from a number of key clinical trials in RT research. An international range of expert authors selected trials for inclusion that have changed clinical practice by influencing national or international guidelines. Selected trials were published in the last 20 years in four common cancer types where RT is a key component of curative intent treatment: breast, lung, urological and lower gastrointestinal. The majority of the included trials were large randomised phase 3 trials. Explanation of some technical terms is included in Box 1 to assist the non-specialist reader.

The collective effect of these clinical trials is that much of the treatment of patients with RT is now based on prospective clinical trial data of efficacy and toxicity rather than historical and empirical practice. There remains, however, considerable progress to be made in further improving outcomes for patients, by ensuring that existing clinical trial evidence is translated into global clinical practice, and by instigating further research into novel technologies and drug–RT combinations.

Box 1 Common radiotherapy terminology **Brachytherapy**: The delivery of radioactive material very close to or within a patient’s tumour to provide high doses of radiation at a short distance. Brachytherapy is also known as sealed source therapy.

**Electrons**: Treatment using an electron beam, usually for skin tumours or superficial tumours.

**External beam therapy**: All types of radiation therapy delivered from outside the patient; includes photons, protons and electrons.

**Fraction**: Radiation therapy is typically split into multiple treatments, known as fractions. These are often given daily over several weeks to enable a tumouricidal dose to be given whilst allowing time for normal tissue recovery.

**Hypofractionation**: Treatment involving a decreased number of fractions of increased fraction size (>2 Gy per fraction).

**Hyperfractionation**: Treatment involving an increased number of fractions of reduced fraction size (<2 Gy per fraction).

**Image-guided RT (IGRT)**: Any radiation therapy that includes imaging pre-treatment or during delivery to improve the accuracy of radiotherapy.

**Intensity modulated radiotherapy (IMRT)**: A precise form of radiation therapy using multiple small fields to closely conform the dose of radiation to the tumour.

**Molecular radiotherapy**: The delivery of radiation to a cancer using radiopharmaceuticals which interact with molecular sites or receptors, for example Radium-223.

**Photons**: High-energy X-ray treatment; comprises the majority of radiation treatment.

**Proton beam radiotherapy**: Treatment using a proton beam.

**Stereotactic ablative RT, stereotactic body RT (SABR, SBRT)**: Highly precise irradiation of an extra-cranial lesion using a high dose of radiation in a small number of fractions.

## Breast RT practice-changing trials

The Early Breast Cancer Trialists’ Collaborative group has clearly demonstrated, through their individual patient-data meta-analyses from randomised clinical trials (RCTs), that RT improves local control and survival following breast-conserving surgery, or following mastectomy for node-positive tumours.^12,13^ The RCTs included in this meta-analysis generally span several decades, with the earliest commencing in 1964 and all trials starting before 2000. More recent practice-changing trials (Table 1) have investigated the use of fewer treatments (hypofractionation), the role of tumour bed boost, partial breast irradiation, and nodal irradiation.

**Table 1:** Summary of breast cancer practice-defining RT clinical trials

Trial name (first author)	Trial methodology	Practice-defining trial results and methods	Publications of trial results	Publications related to trial conduct	Evidence of practice change
Hypofractionation trials					
Canadian hypofractionation trial (Whelan)	Standard whole-breast RT 50 Gy in 25 fractions vs. hypofractionated 42.5 Gy in 16 fractions. Randomised 1234 patients.	Hypofractionated group non-inferior to control.	^15, 131^	NA	International: ASTRO.^21^
START-B (Agrawal)	50 Gy in 25 fractions over 5 weeks vs. 40 Gy in 15 fractions over 3 weeks. Randomised 2215 patients.	40 Gy in 15 fractions non-inferior to 50 Gy in 25 fractions in terms of locoregional relapse. Significantly less late normal tissue effects for 40 Gy in 15 fractions. Standardisation of: - patient position - target volumes - dose and fractionation - prescription points - quality assurance.	^16, 132^	^133– 144^	International: ASTRO.^21^ UK: NICE.^22^ RCR Breast consensus meeting.^145^
Effect of boost dose					
EORTC 22881-10882 boost vs. no boost (Bartelink)	Boost dose of 16 Gy to the primary tumour bed after tumourectomy and 50 Gy whole-breast irradiation vs. no additional boost. Randomised 2661 patients.	Improved local control with boost dose, but no significant effect on survival. Increased late normal tissue toxicity with boost. Improved RT QA for boost techniques. Validation of methodology for cosmetic assessment of breast.	^25, 26, 146^	^147– 151^	International: ESMO.^152^ UK: RCR consensus statement.^145^
Partial breast irradiation					
GEC-ESTRO APBI (Strnad)	Compared several different regimens of accelerated partial breast irradiation (APBI) using brachytherapy vs. whole-breast RT 50 Gy in 15 fractions ± boost. Randomised 1184 patients.	APBI using brachytherapy was non-inferior to whole-breast RT.	^32^	NA	NA
IMPORT LOW (Coles)	Whole-breast radiotherapy (WBRT) 40 Gy in 15 fractions vs. 40 Gy to the tumour bed and 36 Gy to the rest of the breast OR 40 Gy to the tumour bed only. Randomised 2018 patients.	PBI non-inferior to WBRT at 5 years in terms of locoregional recurrence, with a reduction in some late normal tissue toxicity endpoints. Introduction of forward planned intensity modulated RT. BASO guidelines now recommend placement of surgical clips to facilitate accurate post-operative radiotherapy planning.	^33^	^153– 157^	International: Danish Breast Cancer Group national guidelines. UK: RCR consensus statement.^145^
Nodal irradiation					
EORTC 10981-22023 – AMAROS trial (Donker)	Surgical vs. radiation treatment of axilla after positive sentinel lymph node biopsy. Randomised 4823 patients.	Comparable local control results for both treatments (under-powered), decreased lymphoedema with RT. Improved RT QA for axillary RT. Improved surgical QA for SLNB.	^34^	^158, 159^	UK: RCR consensus statement.^145^ Association of Breast Surgeons consensus statement.^160^
EORTC 22922/10925 IMC trial (Poortmans)	Whole-breast/thoracic-wall RT + regional nodal irradiation vs. whole-breast/thoracic-wall RT alone. Randomised 4004 patients.	Addition of regional nodal irradiation improved disease-free survival. Improved RT QA for IMC & medial supraclavicular nodal RT.	^35^	^161– 165^	International: ASCO.^38^
MA20 (Whelan)	Women with node-positive or high-risk node-negative breast cancer: whole-breast irradiation plus regional nodal irradiation vs. whole-breast irradiation alone. Randomised 1832 patients	Addition of regional nodal irradiation to whole-breast irradiation did not improve overall survival but reduced the rate of breast-cancer recurrence.	^36^	NA	International: ASCO.^38^
DBCG -IMN (Thorsen)	Prospective population-based cohort study. Patients with right-sided disease were allocated to IMC RT, whereas patients with left-sided disease were allocated to no IMC RT (risk of radiation-induced heart disease). Included 3089 patients.	IMC RT increased overall survival in patients with early-stage node-positive breast cancer.	^37^	^166^	NA

*ASCO* American Society of Clinical Oncology, *ASTRO* American Society for Radiation Oncology, *BASO* British Association of Surgical Oncology, *ESMO* European Society for Medical Oncology, *NICE* National Institute for Health and Care Excellence, *RCR* Royal College of Radiologists

### Hypofractionation, boost and partial breast RT

Evidence from more than 7000 women treated and followed up for 10 years within the UK START and Canadian RCTs demonstrated that shorter, hypofractionated courses of RT are non-inferior to 5 weeks of ‘conventionally’ fractionated therapy (25 fractions) for local tumour control. Furthermore, these hypofractionated treatments cause not more (Canadian fractionation schedule: 16 fractions of 2.66 Gy) or even fewer (UK fractionation schedule: 15 fractions of 2.67 Gy) late side effects^14–16^ than the conventional regimens. This confers important benefits, since late RT toxicity tends to be permanent.^17^ In addition, fewer RT visits are more convenient for patients and more cost-effective for healthcare providers.^18–20^ These results led to hypofractionation being adopted within international guidelines from the American Society for Radiation Oncology (ASTRO) and the UK National Institute for Health and Care Excellence (NICE).^21,22^ While hypofractionation is now standard practice in the UK and widely adopted in Canada, uptake in the United States and Europe is patchy, despite the convincing evidence. This is perceived to be a consequence of professional and societal barriers to optimal care, together with variations in treatment economics across different healthcare systems and specifically reimbursement, which is still calculated per fraction in many countries.^23,24^

The EORTC 22881-10882 study randomised patients to receive either a boost dose to the tumour bed following whole-breast irradiation, or whole-breast irradiation alone. This trial showed improved local control in patients receiving the boost, although this was at the cost of increased late side effects.^25,26^ After long-term updates, which confirmed the results and enabled better subgroup analyses, this trial led to international guidelines widely recommending a boost for patients ‘at high risk of recurrence’ following breast conserving surgery, but advising that the boost could be safely avoided for those at low/intermediate risk.^21,22^

The aim of partial irradiation is to focus RT on the region at highest risk of recurrence, to maintain high rates of local tumour control while minimising toxicity by virtue of a smaller irradiated volume. Very promising results were demonstrated by the Budapest and Florence partial breast RT trials, but the relatively small numbers in these studies have been insufficient to change practice internationally.^27,28^ Intra-operative partial breast RT has also been investigated and is potentially very attractive to patients if it means post-operative RT can be avoided. However, the ELIOT trial using intra-operative electrons failed to show non-inferiority with whole-breast RT; this may have been at least in part due to patient selection due to inclusion of patients with higher risk of recurrence.^29^ The TARGIT trial using photon intra-operative RT reported non-inferiority with whole-breast RT, but non-standard statistical analysis was used and median follow up was very short at only 2 years 5 months.^30,31^ Two large randomised partial breast RT trials have recently reported: IMPORT LOW and GEC-ESTRO.^32,33^ IMPORT LOW reported that partial breast RT, delivered over an identical 3-week period to the control group, yielded non-inferiority in local relapse rates while also reducing side effects. These results have been published too recently to be labelled ‘practice changing’; however, the study marked an important step in reducing late toxicity for a large population of low-risk patients. IMPORT LOW also used a simple RT technique leveraging existing radiotherapy equipment and standard RT techniques, with the only difference being the reduced treatment volume. The GEC-ESTRO trial, which used a brachytherapy approach for accelerated partial breast irradiation, confirmed non-inferiority with whole-breast RT. The results of four further phase 3 RCTs investigating partial breast RT using different techniques will be published in the next few years (NCT00103181, NCT00282035, NCT01247233, NCT01803958).

### Nodal irradiation

Some breast cancer recurrences occur locoregionally and therefore the local management of local lymph node groups is an important consideration, especially in patients at higher risk of recurrence. The AMAROS trial randomised patients to either surgical dissection or axillary node RT, following positive sentinel lymph node biopsy, and reported reduced rates of lymphoedema in the RT arm with comparable loco-regional tumour control rates. This important study provides patients with more options after a positive sentinel lymph node biopsy, and has led to a dramatic fall in axillary surgery in many centres.^34^ More recently, there has been increasing interest in the addition of internal mammary chain (IMC) nodal irradiation to standard RT. Two large RCTs were published in 2015, both of which showed an improvement in disease-free survival with the addition of IMC irradiation.^35,36^ This was confirmed in a population-based Danish study where patients with right-sided breast cancer received IMC-RT, whereas left-sided patients did not and received normal standard of care at the time.^37^ These studies led to IMC irradiation being re-introduced in the 2016 ASCO post-mastectomy RT guidelines.^38^

Taken together, these practice-changing trials have facilitated ‘risk-adapted RT’, whereby breast RT approaches are offered based on the individual’s broad risk of recurrence. These range from partial breast RT in lower risk patients to IMC RT in high-risk patients in order to optimise local tumour control while minimising side effects. This demonstrates that RT for breast cancer is no longer a ‘one size fits all’ strategy, although we are still some way from delivering truly personalised breast RT. Further research is needed to: reliably identify which group of patients can avoid breast RT completely^39^; determine optimal timing of RT with mastectomy and reconstruction^40^; determine whether there is an advantage to deliver breast RT pre-operatively and how to best combine RT with novel drugs in higher risk patients.^41^

## Lung RT practice-changing trials

In recent years we have seen many technological advances in the field of lung radiotherapy. These include the integration of 4-dimensional computed tomography (CT) and positron emission tomography (PET) for planning; the improved target conformality with the delivery of IMRT; SBRT; and the optimisation of image guidance (Table 2).

**Table 2:** Summary of lung cancer practice-defining RT clinical trials

Trial name (first author)	Trial methodology	Practice-defining trial results and methods	Publications of trial results	Publications related to trial conduct	Evidence of practice change
Early-stage non-small cell lung cancer					
Indiana SBRT (Timmerman)	Phase 2 study of stereotactic body RT (SBRT) for T1/2N0M0 NSCLC in patients unfit for lobectomy. 70 patients.	Excessive toxicity for central tumours; practice changing in developing the ‘No Fly’ zone avoiding central airways for SBRT.	^43, 167^	NA	International: ESMO.^52^
RTOG 0236 (Timmerman)	Phase 2 multicentre study of SBRT for T1/2N0M0 medically inoperable lung cancer. 55 patients.	High rates of local tumour control (>90% at 3 years).	^45^	NA	International: ESMO.^52^ NCCN.^168^
Dutch population-based SABR paper (Palma)	Population-based study investigating the impact of introducing SBRT in 875 patients 75 years of age or older.	SBRT introduction was associated with a decline in the proportion of untreated elderly patients, and an improvement in overall survival.	^53^	NA	International: ESMO.^52^ NCCN.^168^
SPACE (Nyman)	Phase 2. Randomised 102 patients with stage I medically inoperable NSCLC to receive SBRT to 66 Gy in three fractions (1 week) or 3DCRT to 70 Gy (7 weeks).	First randomised study of SBRT compared to conventional dose fractionation. Better local control, similar OS and less toxicity with SBRT.	^169^	NA	International: ESMO.^52^ NCCN.^168^
Locally advanced/metastatic non-small cell lung cancer					
RTOG0617 (Bradley)	Phase 3, 2 × 2 factorial design. Randomised 544 patients to 60 or 74 Gy concurrently with carboplatin/paclitaxel, with or without additional cetuximab.	74 Gy was no better than 60 Gy and potentially harmful. Secondary analysis provided evidence supporting the use of IMRT in lung cancer.	^59, 60^	NA	International: ESMO.^52^
QUARTZ (Mulvenna)	Phase 3. Randomised 538 NSCLC patients with brain metastases to dexamethasone and optimal supportive care with or without whole-brain RT.	No significant difference in QUALYs (primary endpoint), overall survival, overall quality of life or dexamethasone use between the two groups.	^61^	NA	International: ESMO.^52^
Limited-stage small cell lung cancer					
Intergroup0096 (Turrisi)	Phase 3. Randomised 417 patients with LS-SCLC to 4 cycles of chemotherapy cisplatin-etoposide with either once (OD) or twice-daily (BD) radiotherapy.	BD RT showed improved overall survival at 2 and 5 years.	^62^	NA	International: ESMO.^66^ NCCN.^170^
PCI01 -EULINT1 (LePechoux)	Phase 3. Randomised 720 patients with LS-SCLC and complete response after chemotherapy and thoracic RT. Randomised dose of prophylactic cranial irradiation (PCI) to standard (25 Gy in 10#) vs. higher dose (36 Gy in 18# or 36 Gy in 24# given BD).	No significant reduction in brain metastases at 2 years with higher dose PCI. 25 Gy in ten fractions to remain standard-of-care. Standardisation of PCI dose in limited-stage SCLC internationally.	^171^	NA	International: ESMO.^66^
CONVERT (Faivre-Finn)	Phase 3. Randomised 547 patients with LS-SCLC to twice-daily (45 Gy in 30 fractions) vs. once-daily (66 Gy in 33 fractions) concurrently with chemotherapy.	OD RT did not result in a superior survival compared to BD RT.	^63^	^172– 174^	NA
Extensive-stage small cell lung cancer					
EORTC prophylactic cranial irradiation trial (Slotman)	Phase 3. Randomised 286 patients to PCI vs. no further treatment in patients with extensive small cell lung cancer who had responded to chemotherapy.	Significant improvement in median overall survival with PCI, as well as a lower cumulative risk of brain metastases at 1 year.	^64^	^175^	International: ESMO.^66^ NCCN.^170^
CREST (Slotman)	Phase 3. Randomised 498 patients to thoracic radiotherapy 30 Gy in ten fractions vs. no thoracic radiotherapy, in patients with extensive-stage small cell lung cancer who had responded to chemotherapy.	No significant difference in 1-year survival (primary endpoint). Significant improvement in overall survival at 2 years with thoracic radiotherapy, with no increase in severe (grade 3 or higher) toxicity.	^65, 176^	^177, 178^	International: Survey of European centres: 81% now giving thoracic RT in ES-SCLC compared to 25% previously.^177^ NCCN.^170^

*ESMO* European Society for Medical Oncology, *NCCN* National Comprehensive Cancer Network

### Early-stage non-small cell lung cancer

A key change in lung RT has been the introduction of SBRT for early-stage non-small cell lung cancers (NSCLC). This development came after multiple studies investigated this highly targeted and high-dose RT, which is delivered in just a few fractions. Treatment outcomes are comparable with surgery, especially for patients with medical co-morbidities.^42–51^ Evidence-based guidelines, mostly based on non-randomised phase II trials, distinguish patients who can be treated safely with SBRT from those at risk of excessive toxicity (especially those with centrally located tumours). Patients at higher risk of toxicity should be treated with a dose-adapted SBRT regimen, preferably within further clinical trials to allow collection of high-quality prospective toxicity data.^52^ Population-based studies have also demonstrated the effectiveness of this technique for improving overall survival in a non-selected elderly patient population.^53,54^

### Locally advanced NSCLC

One of the main strategies for improving outcomes in patients with locally advanced NSCLC is dose escalation, which has shown encouraging results in phase I–II trials over the last two decades.^55–58^ However, the outcome of the phase III Radiation Therapy Oncology Group (RTOG) 0617 trial was surprisingly disappointing;^59^ dose escalation with concurrent chemoradiotherapy to 74 Gy in 37 fractions led to worse survival, compared with the standard of care (60 Gy in 30 fractions). The results of RTOG 0617 had a profound impact on usual clinical practice and future clinical trials, establishing 60 Gy in 30 fractions as the new benchmark for chemoradiotherapy in locally advanced NSCLC. Importantly, it also provided prospective evidence supporting IMRT for NSCLC, as a secondary analysis showed IMRT produced lower rates of severe pneumonitis and resulted in lower cardiac doses, compared with conventional RT.^60^

### NSCLC and brain metastases

Avoidance of unnecessary treatment is important, especially for patients requiring palliation. The QUARTZ trial examined the role of whole-brain RT (WBRT) plus optimal supportive care in patients with NSCLC and brain metastases. The trial found no improvement in survival or quality adjusted life years with the addition of WBRT.^61^ As a result, WBRT is usually not recommended for the majority of patients; however, it may still be beneficial for some patients with better prognosis, such as those with driver mutations.

### Small cell lung cancer

Very little progress was made for several decades in the systemic treatment of both limited- and extensive-stage small cell lung cancer (SCLC). Recently, advances in RT techniques, use of prophylactic cranial irradiation for all stages of SCLC, and improved combination of chemotherapy with RT have led to major improvements in survival. The current standard of care for patients with limited-stage SCLC is based on an RCT that compared once daily with twice-daily RT delivered concurrently with chemotherapy, which demonstrated superiority of twice-daily RT in terms of survival.^62^ However, since the publication of this study in 1999, there has been a lack of consensus regarding routine use of twice-daily RT, despite its superiority, due to logistical issues and concerns regarding toxicity (for example, one-third of the patients developed ≥grade 3 radiation oesophagitis). To help resolve this, the CONVERT trial compared twice-daily RT (45 Gy in 30 fractions) to a higher RT dose delivered once daily (66 Gy in 33 fractions), both given concurrently with chemotherapy.^63^ Overall survival outcomes did not differ between the two groups; however, the survival achieved in both groups was higher and toxicity much lower (>50% reduction) than previously reported.^62^ As this trial was designed to show superiority of once-daily RT and was not powered to show equivalence, the implication is that twice-daily RT should be considered the standard of care.

In the extensive-stage setting, an EORTC trial demonstrated that prophylactic cranial irradiation in patients who had responded to chemotherapy reduced the incidence of brain metastases and improved survival, compared with no subsequent treatment.^64^ Later, the CREST trial randomised extensive-stage SCLC patients to receive either thoracic RT (30 Gy in ten fractions) and prophylactic cranial irradiation, or to receive prophylactic cranial irradiation alone. Although there was no difference in overall survival at 1 year, a pre-planned analysis revealed a significant improvement in overall survival at 2 years, with a low rate of severe toxicities.^65^ Both of these trials were practice changing, and have led to new recommendations in international guidelines.^66^

The studies discussed have demonstrated significant survival improvements in both NSCLC and SCLC patients. Areas of unmet research need include the evaluation of modern RT technologies (such as SBRT and protons) in a wider population, and the development of individualised treatment strategies.

## Urological RT practice-changing trials

Radiation-based therapy is used as an alternative to radical prostatectomy for localised disease, producing equivalent survival to surgery.^67^ It is also used as the key treatment modality for locally advanced disease. The development of RT as a curative treatment has been supported by technical refinement of RT, including treatment delivery with reduced toxicity, dose escalation and use of concomitant androgen deprivation therapy (ADT) (Table 3).

**Table 3:** Summary of urological cancer practice-defining RT clinical trials

Trial name (first author)	Trial methodology	Practice-defining trial results and methods	Publications of trial results	Publications related to trial conduct	Evidence of practice change
Prostate					
PR-07 (Mason)	Androgen deprivation therapy (ADT) alone vs. ADT + RT in men with T3-4N0M0 prostate cancer, or T1-2 disease with PSA > 40 or PSA 20–40 with Gleason score 8–10. Randomised 1205 patients.	Overall survival significantly improved by the addition of RT.	^69^	NA	UK: NICE.^179^
SPCG-7 (Widmark)	ADT alone vs. ADT + RT. Randomised 875 patients.	Addition of RT improved overall survival and prostate cancer specific mortality.	^68^	NA	UK: NICE.^179^
MRC RT-01 (Dearnaley)	Radical RT 64 Gy in 32 fractions vs. 74 Gy in 37 fractions. Randomised 843 patients.	Significantly improved biochemical progression-free survival in the 74 Gy dose group, but no improvement in overall survival.	^72, 73^	^180^	International: NCCN.^181^ UK: NICE.^179^
Dutch (Peeters)	78 Gy vs. 68 Gy in patients with T1-4 prostate cancer with PSA < 60. Randomised 669 patients.	Significantly improved biochemical progression-free survival for 78 Gy but no effect on overall survival. Higher rates of acute and late GI and GU toxicity for 78 Gy group.	^71, 182^	NA	International: NCCN.^181^ UK: NICE.^179^
RTOG 0415 (Lee)	73.8 Gy in 41 fractions vs. a hypofractionated regime 70 Gy in 28 fractions for men with low-risk prostate cancer. Randomised 1092 patients.	Hypofractionated regime non-inferior to conventional fractionation, but resulted in significantly increased late grade 2/3 GI and GU toxicity.	^80^	NA	NA
HYPRO (Aluwini)	Hypofractionated RT of 64·6 Gy in 19 fractions, three fractions per week vs. 78 Gy in 39 fractions, five fractions per week, in men with intermediate-high-risk prostate cancer. Randomised 804 patients.	Hypofractionated regime was not superior to the conventionally fractionated regime in terms of 5-year relapse-free survival, with higher incidence of acute GI toxicity, late GI and late GU toxicity.	^79, 183, 184^	NA	NA
CHHiP (Dearnaley)	60 Gy in 20 fractions OR 57 Gy in 19 fractions) vs. standard dose 74 Gy in 37 fractions for radical treatment of T1-3aN0M0 prostate cancer with PSA < 30. Randomised 3216 patients.	60 Gy in 20 fractions over 4 weeks non-inferior to conventionally fractionated radiotherapy, with similar rates of toxicity. Dose constraints designed for the CHHiP trial adopted in other trials. Supported implementation of IMRT for prostate cancer treatment.	^75, 76^	NA	International: AUA/ASTRO/SUO: guideline.^185^ German Society of Radiation Oncology guideline.^186^ UK: RCR dose fractionation guideline.^77^
PROFIT (Catton)	78 Gy in 39 fractions vs. hypofractionated RT of 60 Gy in 20 fractions over 4 weeks, with both arms receiving no ADT. Randomised 1206 men.	Non-inferior biochemical-clinical failure for hypofractionation with no increase in grade 3 or higher late GI or GU toxicity.	^81^	NA	International: AUA/ASTRO/SUO: guideline.^185^ German Society of Radiation Oncology guideline.^186^ UK: RCR dose fractionation guideline.^77^
ALSYMPCA (Parker)	Radium-223 vs. placebo in men with castration-resistant prostate cancer and bone metastases, given 4 weekly for a total of 6 injections. Randomised 921 patients in a 2:1 ratio.	Improved overall survival for radium-223, with longer time to first symptomatic skeletal related event and improved quality of life scores in the radium group.	^82, 83, 187^	NA	International: ESMO.^84^ NCCN.^181^ UK: NICE.^85^
Bladder					
BC2001 (James)	ChemoRT (5FU/mitomycin C) vs. RT alone; patients also randomised to receive whole-bladder or modified volume RT. Randomised 360 patients.	Improved locoregional control of bladder cancer with chemoRT, without a significant increase in adverse events. Dose constraints derived from BC2001 data used in IDEAL, HYBRID and RAIDER studies.	^86, 188^	^189^	UK: NICE.^88^
BCON (Hoskin)	RT alone vs. RT plus carbogen-nicotinamide (CON) in locally advanced bladder cancer. Randomised 333 patients.	Overall survival significantly improved with the addition of CON.	^87^	NA	UK: NICE.^88^

*AUA/ASTRO/SUO* American Urological Association/American Society for Radiation Oncology/Society of Urologic Oncology, *ESMO* European Society for Medical Oncology, *NCCN* National Comprehensive Cancer Network, *NICE* National Institute for Health and Care Excellence, *RCR* Royal College of Radiologists

### Localised prostate cancer

RCTs have led to a significant improvement in the evidence base supporting RT for localised prostate cancer over the last two decades. Prior to this, clinical practice was largely based on non-randomised studies. Recognition of the benefit for local control, even in high-risk/locally advanced patients, has been of key importance. The PR07 trial in locally advanced or ‘high risk’ localised node-negative prostate cancer provided evidence for a clear survival benefit of RT in addition to ADT, and similar results were reported by a Scandinavian trial.^68,69^ Non-randomised data from the STAMPEDE RCT also showed significantly improved survival with combined RT and ADT, compared with ADT alone, in patients with both high-risk and node-positive prostate cancer.^70^ Furthermore, several randomised dose-escalation studies have provided evidence to support an increased RT dose from 64–68 Gy to 74–78 Gy.^71–73^ These dose escalation trials showed improvements in biochemical progression-free survival, but not overall survival. The addition of RT to ADT, and use of a higher dose range 74–78 Gy, are now routine practice in the UK. Recent data suggest that additional benefit could be accrued through further dose escalation using a brachytherapy boost in high-risk patients, at the cost of increased toxicity.^74^

Based on the principle that prostate cancer may be more sensitive to increases in daily dose per fraction than previously thought, the CHHiP trial compared two hypofractionated regimens (60 Gy in 20 fractions vs. 57 Gy in 19 fractions) to the standard UK regimen at the time (74 Gy in 37 fractions), in men with T1b–T3aN0M0 prostate cancer. The 5-year results demonstrated that 60 Gy in 20 fractions was non-inferior to the standard regimen in terms of biochemical or clinical failure, and was associated with similar toxicity.^75,76^ As a result, this trial has already changed practice in the majority of UK centres.^77,78^ The RTOG 0415, HYPRO and PROFIT trials have also recently published data supporting the use of moderate hypofractionation; it seems likely that these trials together will lead to wider international use of hypofractionation in low-intermediate-risk prostate cancer.^79–81^

### Metastatic prostate cancer

The most common metastatic site in prostate cancer is bone. Delivery of RT to multiple sites of bone disease can be achieved by using bone-seeking radiopharmaceuticals. Beta-emitting radioisotopes such as strontium-89 have been used in the palliation of bone pain for many years. More recently the alpha-emitting radioisotope radium-223, given as a course of 6-monthly injections, has been shown to be a key addition to the treatment options for men with castration-resistant prostate cancer with bone metastases. The ALSYMPCA trial clearly demonstrated an improvement in overall survival, quality of life scores, and time to first symptomatic skeletal-related event for radium-223, when compared with placebo.^82,83^ This treatment has been rapidly included in international guidelines and clinical practice, and provides another important addition to treatment options for men with castration-resistant prostate cancer.^84,85^

### Muscle-invasive bladder cancer

RT has been used as an alternative bladder-preserving modality to radical cystectomy for muscle-invasive bladder cancer, but has been limited by the lower rate of complete response. To address this issue, over the last two decades the addition of either chemotherapy or radiation sensitisers to RT has been explored to improve local control. The evidence for these two treatment options has been has been provided by two randomised trials: the BC2001 trial showed an improvement in locoregional disease-free survival with the addition of 5-fluorouracil (5FU) and mitomycin C to RT,^86^ and the BCON trial showed improved overall survival with the addition of nicotinamide and carbogen (a gas mixture of 2% carbon dioxide and 98% oxygen).^87^ The low recurrence rates observed in these trials have led to growing acceptance that RT with concomitant chemotherapy or radiation sensitisers is a valid alternative to cystectomy for many patients, and led to NICE recommending that either RT or surgery should be offered to patients being treated with curative intent.^88^

## Lower gastrointestinal RT practice-changing trials

Radical surgical resection is the cornerstone of treatment for localised rectal cancer; however, in the 1980s, radical surgery alone resulted in unacceptably high rates of local recurrence.^89^ Significant improvements in surgical techniques, such as total mesorectal excision (TME), resulted in lower local recurrence rates and led to a range of phase III trials that tested the additional benefit of pre-operative RT (Table 4).

**Table 4:** Summary of colorectal cancer practice-defining RT clinical trials

Trial name (first author)	Tumour site	Trial methodology	Practice-defining trial results	Publications of trial results	Publications related to trial conduct	Evidence of practice change
Short course hypofractionated radiotherapy trials in rectal cancer						
Dutch TME Trial (Kapiteijn)	Rectal	Pre-operative short course radiotherapy 25 Gy in five fractions followed by total mesorectal excision (TME) surgery vs. TME surgery alone. Randomised 1861 patients.	Reduced local recurrence rates. No difference in overall survival.	^90, 190^	^191– 199^	International: ESMO.^92^ NCCN.^200^ UK: NICE.^97^
MRC CR07 (Sebag-Montefiore)	Rectal	Pre-operative short course radiotherapy 25 Gy in five fractions followed by surgery vs. surgery with highly selective post-operative chemoradiotherapy 45 Gy in 25 fractions with concurrent fluorouracil. Randomised 1350 patients.	Reduced local recurrence rates and improved disease-free survival in the pre-operative radiotherapy arm. No difference in overall survival.	^91^	^201, 202^	International: ESMO.^92^ NCCN.^200^ UK: NICE.^97^
Long-course radiotherapy ± concurrent chemotherapy trials rectal cancer						
EORTC 22921 (Bosset)	Rectal	Pre-operative RT vs. pre-operative chemoRT vs. pre-operative RT and post-operative chemotherapy vs. pre-operative chemoRT and post-operative chemotherapy in locally advanced rectal cancer. Radiotherapy 45 Gy in 25 fractions. Concurrent chemotherapy 5FU and leucovorin D1-5, 29–33. Randomised 1011 patients.	Reduced local recurrence when concurrent 5FU/LV was added in the first and fifth week of radiotherapy. No difference in overall survival.	^93^	NA	International: ESMO.^92^ NCCN.^200^ UK: NICE.^97^
EORTC 9203 (Gerard)	Rectal	Pre-operative RT vs. pre-operative chemoRT. Randomised 733 patients.	Reduced local recurrence when concurrent 5FU/LV was added in the first and fifth week of radiotherapy. No difference in overall survival.	^94^	NA	International: ESMO.^92^ NCCN.^200^ UK: NICE.^97^
German Rectal Cancer Trial (Sauer)	Rectal	Pre-operative CRT (50.4 Gy) with post-operative CRT (55.8 Gy) with concurrent 5FU infusion weeks 1 and 5. Randomised 823 patients.	Reduced local recurrence, acute and late toxicity with pre-op CRT. No difference in overall survival.	^95^	NA	International: ESMO.^92^ NCCN.^200^ UK: NICE.^97^
NSABP R03 (Roh)	Rectal	Pre-operative CRT (50.4 Gy) with post-operative CRT (50.4 Gy) with concurrent 5FU LV D1-5, 29–33 in locally advanced rectal cancer. Randomised 267 patients.	Improved disease-free survival in favour of pre-op CRT.	^96^	NA	International: ESMO.^92^ NCCN.^200^ UK: NICE.^97^
Short-course hypofractionated vs. long-course concurrent chemoradiotherapy trials in rectal cancer						
Polish trial (Bujko)	Rectal	Pre-operative short course RT (25 Gy in five fractions) with pre-op CRT (50.4 Gy) and 5FU LV D1-5 and 29–33. Randomised 312 patients.	Comparable local recurrence overall survival and toxicity for the two treatment regimens.	^99^	NA	International: ESMO.^92^ UK: NICE.^97^
TROG (Ngan)	Rectal	Pre-operative short course RT (25 Gy in five fractions) with pre-op chemoRT (50.4 Gy) and 5FU LV D1-5 and 29–33. Randomised 312 patients.	Comparable local recurrence overall survival and toxicity for the two treatment regimens.	^100^	NA	International: ESMO.^92^ UK: NICE.^97^
Concurrent and additional cisplatin in anal cancer						
ACT-2 (James)	Anal	Fluorouracil with mitomycin (MMC/FU), to fluorouracil with cisplatin, with or without maintenance doses at weeks 11 and 14. Randomised 940 patients.	No improvement in 3-year progression-free survival or in complete response rates by substituting mitomycin for cisplatin, nor by adding maintenance therapy. ACT2 used a shrinking field two phase protocol and this become standard radiotherapy practice in the UK.	^107, 203^	^204, 205^	International: ESMO.^104^ NCCN.^206^
RTOG 98-11 (Ajanani)	Anal	Neoadjuvant and concurrent cisplatin 5FU vs. concurrent Mitomycin C and 5FU in patients with anal cancer. Randomised 644 patients.	MMC 5FU CRT remains the standard of care. Neoadjuvant and concurrent cisplatin and 5FU resulted in inferior outcomes disease-free and overall survival.	^105, 207^	^208, 209^	International: ESMO.^104^ NCCN.^206^
ACCORD 03 (Peiffert)	Anal	Standard vs. high-dose boost and with or without neoadjuvant cisplatin 5FU chemotherapy. Randomised 307 patients.	Higher dose boost and neoadjuvant chemotherapy did not improve cancer outcomes.	^106^	NA	International: NCCN.^206^
Optimising chemoradiotherapy for anal cancer						
EXTRA (Glynne-Jones)	Anal	Single arm phase II trial of Mitomycin C capecitabine and radiotherapy (50.4 Gy in 28 F). Entered 31 patients.	Acceptable rates of acute toxicity and radiotherapy compliance. Showed that capecitabine can be used instead of 5U with MMC and RT.	^108^	NA	International: ESMO.^104^ NCCN.^206^
RTOG 0529 (Kachnic)	Anal	Single arm phase II trial if IMRT (54 Gy in 28 F to GTV and 42 Gy in 28 F to CTV) with Mitomycin C and 5FU. Entered 63 patients.	Effective and well-tolerated treatment regimen. Supported the introduction if IMRT for a rare cancer.	^109^	NA	International: ESMO^104^

*ESMO* European Society for Medical Oncology, *NCCN* National Comprehensive Cancer Network, *NICE* National Institute for Health and Care Excellence

### Pre-operative RT in rectal cancer

Two phase III international trials demonstrated statistically significant reductions in local recurrence when 25 Gy in five fractions was given prior to TME, compared with TME alone.^90,91^ There was no difference in overall survival. These trials changed the standard of care for patients with resectable rectal cancer, with clinical adoption initially occurring in Northern Europe; however, this approach is now globally supported by international guidelines.^92^

Investigating a different strategy, four phase III trials established pre-operative concurrent chemoradiation as a standard of care for locally advanced rectal cancer. Two of these trials demonstrated that addition of concurrent 5FU and leucovorin to RT (45 Gy in 25 fractions) significantly reduced local recurrence, with no difference in overall survival.^93,94^ Two additional phase III trials demonstrated reduced local recurrence and toxicity when pre-operative chemoradiation was used, compared with post-operative chemoradiation^95,96^ and this approach is supported in both resectable and locally advanced rectal cancer. The use of pre-operative chemoradiation is now supported by international guidelines.^97,98^

With these two overlapping standards of care, two further phase III trials were performed that directly compared the two approaches.^99,100^ These trials reported no significant difference in local recurrence and survival, and as a result, both approaches are supported by international guidelines. Chemoradiation is recommended as the preferred option when the patient has locoregional disease with very close margins of surgical excision.

### Anal cancer RT practice-changing trials

Three phase III trials performed in the 1980s resulted in a major change in clinical practice.^101–103^ Prior to this, radical surgery was performed that resulted in permanent colostomy. The trials determined concurrent mitomycin C, fluorouracil and RT as the standard of care, resulting in the avoidance of major surgery in the majority of patients. Subsequently the UK ACT2 trial introduced a continuous shrinking-field RT technique, using a lower total dose (50.4 Gy) where initial wide field irradiation was immediately followed by boost radiotherapy targeting areas of visible cancer. This approach was adopted into European practice and supported in European guidelines.^104^ Three phase III trials (ACT2, RTOG 9811 and ACCORD 03) demonstrated that additional chemotherapy either before or after concurrent chemoradiation did not improve cancer outcomes.^105–107^ Furthermore, in the RTOG 9811 trial, pre-operative and concurrent cisplatin-5FU led to higher colostomy rates and inferior disease-free survival rates. In addition, two small phase II trials had a significant effect on clinical practice. The UK EXTRA trial reported acceptable local control and toxicity using concurrent capecitabine rather than 5FU,^108^ and the use of concurrent capecitabine is now supported in international guidelines. The RTOG 0529 phase II trial^109^ reported an improvement in acute toxicity and acceptable outcomes with IMRT, compared with the RTOG 9811 trial, and IMRT is now widely used in anal cancer. It is clear that practice-changing and practice-defining clinical trials are feasible in this rare cancer.

## There is substantial inequality in access to evidence-based RT

Clinical trials have some perceived disadvantages. One such shortcoming is the length of time taken to produce mature results, which is often many years; aspects of management potentially become out-dated by the time the trial is able to report results. Another disadvantage concerns the selective populations that are often treated within trials, which may not represent ‘real life’ patients; patients in trials are typically younger and fitter, have less co-morbidities and are submitted to stricter follow-up schemes than the general patient population.^110^ Both of these aspects can limit uptake of trial results into clinical practice,^111^ and this may be hampered further by infrastructural, organisational or financial barriers, such as those observed for twice-daily RT in treating limited-stage SCLC.

**Fig. 2:**
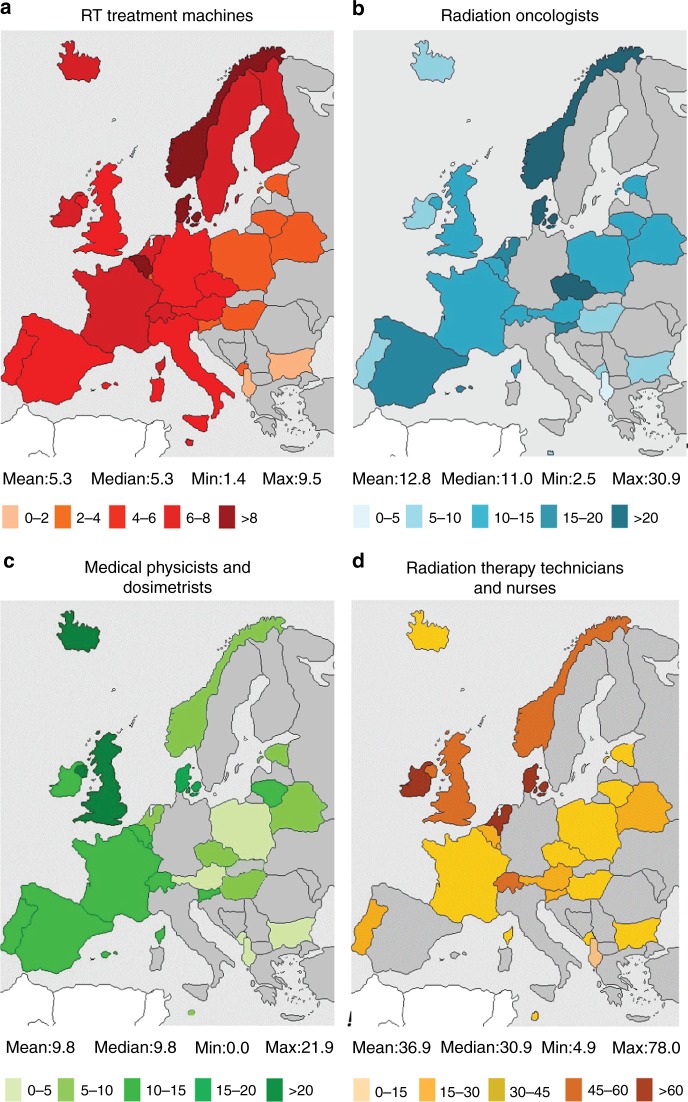
Megavoltage RT equipment and personnel per million inhabitants across Europe. Countries coloured grey indicate no available data. Reprinted from ref. ^130^. Copyright (2015), with permission from Elsevier

The European SocieTy for Radiotherapy and Oncology-Health Economics in Radiation Oncology (ESTRO-HERO) project surveyed European countries regarding the availability of RT facilities and personnel, which are key issues for RT access (Fig. 2). The study reported large variation across Europe in resource availability, and showed a clear correlation with national income. It also highlighted serious gaps in RT provision and staffing that was more pronounced in, although not limited to, countries in Southern and Eastern Europe.^112,113^ Across 40 European countries, RT utilisation was much lower than estimated; just under half were treating less than 70% of patients requiring RT.^114^ Similarly, the Global Task Force on Radiotherapy in Cancer Care and Control (GTFRCC) published a model of worldwide RT supply and demand based on cancer incidence, number of RT machines available and evidence-based guidelines for RT. At least 36 countries had no RT machines at all, and there was a distinct correlation between national income and availability of RT machines. Using available evidence from world-wide clinical trials and guidelines, this report also provided compelling evidence that investment in RT would save many millions of life-years and also create positive economic benefits^4,115^; even if a patient is no longer able to contribute to the workforce, the intrinsic personal or societal value of a life-saving intervention has been estimated to be 2.3 times the gross domestic product per person in a given year.^116^ In order to achieve the ultimate goal of equal access to radiotherapy worldwide, the GTFRCC has set forward a number of ‘Calls to Action’, including measurable targets for expansion of human resources and RT capacity (both requiring sustainable financing), aligning RT access with universal healthcare coverage and, last but not least, systematically including radiotherapy expansion in national cancer control planning.

### How can the RT community tackle these issues?

**Fig. 3:**
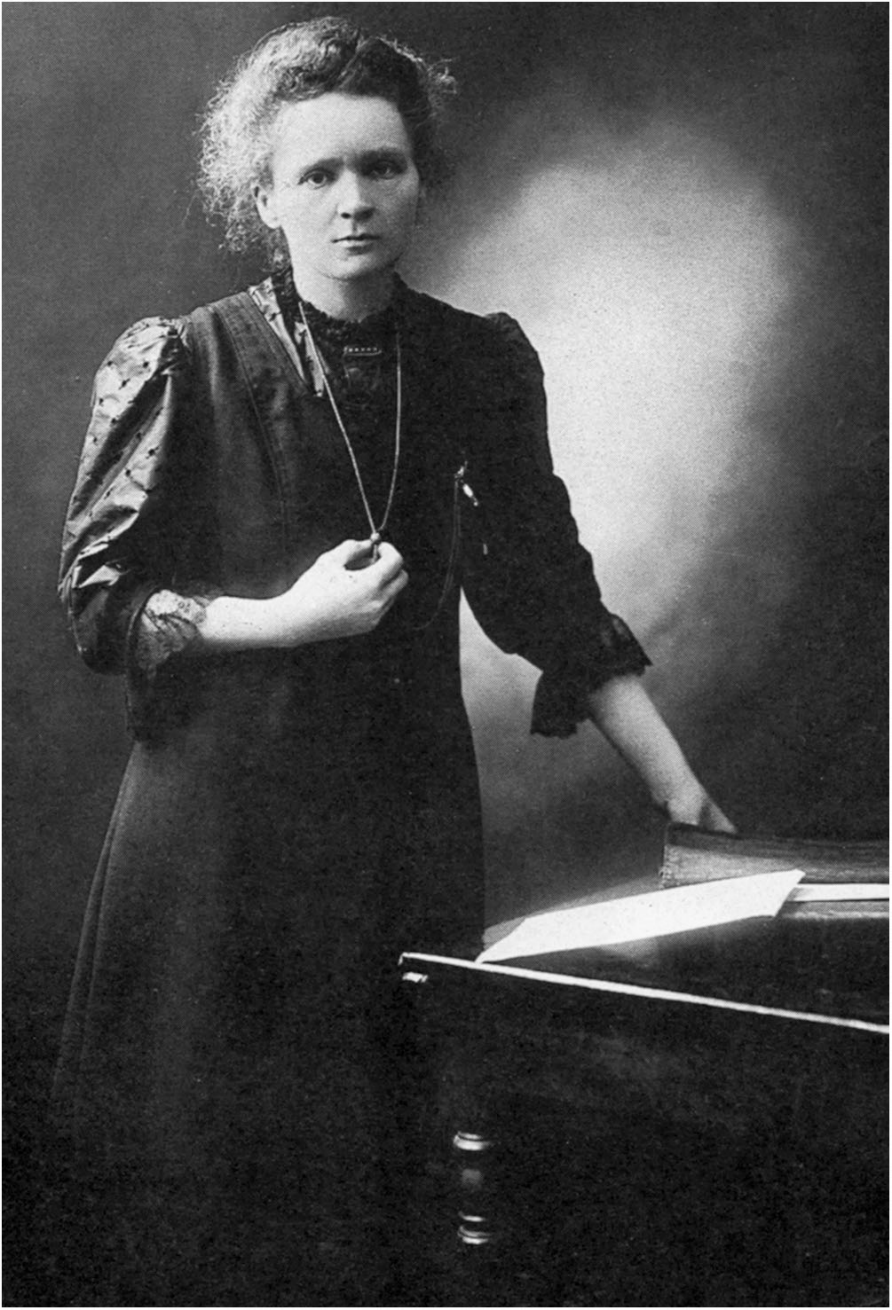
Celebrating 150 years since the birth of Marie Curie. Reproduced with permission from ACJC-Musée Curie

Large-scale clinical trials have provided a solid rationale for making changes in clinical practice to improve patient care; adequate infrastructure and human resources are now needed to enable the implementation of these changes globally. International organisations are working to improve access to RT and to encourage research. The International Atomic Energy Agency (IAEA) has highlighted inequity in access to RT, and it promotes the setting up and maintaining of RT services in low- and middle-income countries through partnerships with centres in high-income countries. ESTRO promotes evidence-based RT and addresses the inequity of access to high-quality RT across Europe. The newly established ESTRO Cancer Foundation aims to raise awareness of RT benefits and to create a community of supporters. Its first initiative is the Marie Curie Legacy Campaign (www.150yearsmariecurie.org), which will educate the general public about the life and work of this iconic scientist, and highlight RT as a key component of cancer treatment (Fig. 3). In addition, the UK Clinical and Translational Radiotherapy Research Working Group (CTRad) facilitates the development of new RT trials and raises awareness of the importance of RT research to funding bodies. A particular success has been through engagement with pharmaceutical companies to drive forward research into RT and novel drug combinations.^11^ Both ESTRO and CTRad are committed to tackling major challenges for future RT trials and assisting stakeholders in developing solutions (Table 5).

**Table 5:** Challenges and solutions for future RT clinical trials

Key challenges for future RT trials
Limited academic funding
Limited pool of clinical academics/academic physicists
Competition with pharmaceutical company-funded trials
Slow accrual of academic RT trials
Lack of studies evaluating novel RT technologies
Little success in the field of drug–RT combination
Possible solutions
Academic groups to lobby funders and mentor the next generation of academics
Improve collaboration between academic groups to improve clinical trial efficiency
Improve collaboration with pharmaceutical industry
Improve methodology of combination studies/evaluation of new technologies to make them more efficient
Improve collaboration between pre-clinical and clinical researchers in the field of drug–RT combination

## What is the future of RT research?

Future RT research will focus on reducing treatment toxicity and on further improving survival rates and management of locoregional relapse. This is likely to be increasingly driven by biomarkers to assist in individualising RT treatment. There now exist validated biomarkers for some cancer types, for example p16 positivity predicts better prognosis and response to chemoRT treatment in head and neck cancer. However, for most cancer types, biomarkers for response to RT treatment and toxicity are not yet established, and this will be an important area of future translational RT research. In this section, we discuss selected areas of predicted future clinical RT research. As we look to further refine RT, our trial designs will also need to evolve accordingly.

### Reducing the toxicity of RT

RT-related toxicity may be reduced by avoiding unnecessary treatment and by continued improvements in tumour targeting, to reduce the ‘safety margin’ of normal tissue around the cancer. Technological developments such as proton beam therapy and the integration of magnetic resonance imaging (MRI) technology into RT are likely to be important areas of future research aiming to improve tumour targeting.

Proton beam therapy has the potential to dramatically reduce doses to nearby critical structures, because the physical properties of the proton radiation beam are very different to photons. This is especially important, for example, near the eye and parts of the central nervous system. It also reduces the risks of ionising radiation in paediatric patients, which can have devastating effects on growth and development.^117^ Until now, proton beam therapy has mainly been introduced in privately funded centres rather than through comparative clinical trials, and thus the evidence base for some tumour types is very limited. Small-scale clinical trials and patient registries are now being established, and it is hoped that the wider introduction of proton beam therapy on a global scale will provide opportunities for larger international clinical trials to demonstrate clinical benefit and assess the cost-effectiveness of this new therapy for a broad range of tumour types.

MRI allows for greater resolution of soft tissue than CT, which means many tumours, such as abdominopelvic cancers, can be identified more accurately, meaning smaller normal tissue margins are needed. MRI is currently widely used to improve the accuracy of radiological tumour staging and to guide the contouring of structures for RT planning, prior to treatment. The current challenge concerns how to accurately identify and monitor these tumours online during treatment. Many centres have the capability to perform daily image guidance with repeat CT scanning before each RT treatment, using the linear accelerator itself; however, this is of limited use for accurately imaging soft tissue, especially where there is considerable organ motion. Recently, hybrid RT machines have been developed that combine a linear accelerator with an MRI scanner (MR-linac) that is able to perform a new generation of imaging before and during RT delivery. This allows accurate localisation of the tumour pre-treatment and supports the ability to adapt treatment daily if required and to monitor the tumour and critical organ movement during treatment.^118,119^ This, in the future, will allow ‘gating’; the automatic switching on and off of the treatment beam according to pre-set parameters, for example to allow for respiratory and bowel motion. This may facilitate greatly improved image guidance of RT delivery, minimising the dose to normal tissues and enabling dose escalation to the tumour.

Assessment of these new technologies will be facilitated by the recently published framework for evaluation of RT technology, R-IDEAL,^120^ and by international clinical and research networks, for example the European Particle Therapy Network (an ESTRO taskforce) and the ATLANTIC MRL research consortium. Recently funded clinical trials are increasingly including imaging, blood or tissue biomarkers, and we expect this will support a move towards the increased personalisation of RT, for example identifying which patients are likely to benefit from RT and tailoring the dose or fractionation. Trial designs that encompass biomarker discovery or prospective validation of predictive biomarkers will maximise value for trial funders and research opportunities for patients.^121,122^

### Improving survival rates with RT

Improved survival rates are likely to occur through employing/adopting RT in novel situations. There is growing interest in using SBRT to deliver a much higher dose than traditionally given for oligometastatic or oligoprogressive disease^123^; early trials have shown improvements in progression-free survival for this approach.^124^ Larger trials aiming to evaluate the role and benefit of ‘radical’ high-dose RT in oligometastatic disease across disease sites are currently underway in the UK (CORE) or in development (ESTRO/EORTC OligoCare studies). SBRT and/or MR-guided RT may also prove beneficial in cancers not traditionally amenable to RT, such as renal and pancreatic cancers, due to the previous inability to irradiate without including large normal tissue margins that precluded the delivery of a tumouricidal dose.

Survival may also be improved through dose escalation via improved image guidance (as previously discussed), and novel drug combinations with RT. There is currently particular interest in combining RT with DNA damage response inhibitors and immunotherapy. RT induces direct cell death by causing single- and double-stranded DNA breaks. Double-stranded DNA breaks are much more lethal to cells than single-strand breaks; however, single-stranded DNA breaks are a more frequent consequence of ionising radiation. DNA damage response inhibitors may therefore act in a ‘synthetically lethal’ manner to selectively increase the number of double-stranded DNA breaks in irradiated cells, with minimal effects on nearby non-irradiated cells.^125^ RT also promotes immune-mediated cell death through the increased release of tumour antigens, the induction of inflammatory cytokines, and the transient overexpression of cell surface receptors.^126^ Together, these mechanisms facilitate effector T cell killing of irradiated tumour cells and priming of antigen-presenting cells to increase the adaptive immune response against tumour cells elsewhere in the body, in non-irradiated areas. There are multiple ongoing clinical trials of DNA damage response inhibitors with RT^127^ and immunotherapy with RT.^128,129^ Efficient trial design in this field, through use of adaptive model-based phase I dose-finding strategies, risk stratification, biomarkers and/or appropriate intermediate endpoints, and multi-arm multi-stage studies will support more rapid evaluation of the most promising drug–RT combinations.

## Conclusion

Major steps have been made in developing new RT techniques and regimens to optimise cancer outcomes, whilst simultaneously minimising toxicity. These have been achieved through high-quality clinical trials, involving collaboration across countries.

Particular successes have been the introduction of SBRT for early-stage NSCLC especially for medically inoperable patients, and the increasing use of hypofractionation in breast and prostate cancer as this improves convenience for patients and cost-effectiveness for healthcare providers, whilst maintaining excellent cancer outcomes. Despite these achievements, and together with the high cost-effectiveness of RT, access to evidence-based RT is not available to everyone. Organisations such as the IAEA, ESTRO and CTRad are making great progress in raising awareness of this inequity and educating both health professionals and policy makers. As we mark the 150th anniversary of Marie Curie’s birth, we hope that international co-operation of the RT community will facilitate high-quality evidenced-based RT for the millions of people who require it, regardless of where they live.
